# Copper Peroxide-Loaded Dual-Responsive Hydrogel for Enhanced Diabetic Wound Healing Through Oxygen Release and Promotion of Angiogenesis

**DOI:** 10.3390/gels12100864

**Published:** 2026-09-24

**Authors:** Lanqin Yu, Xiang Jia, Lejia Xu, Xiaojun Li, Na Li, Lihua Li, Yifan Zhang, Changren Zhou

**Affiliations:** 1 College of Chemistry and Materials Science, Engineering Research Center of Artificial Organs and Materials, Jinan University, Guangzhou 510632, China; ylq06060604@163.com (L.Y.); jx18754297280@163.com (X.J.); m13173025825@163.com (L.X.); xj13592809047@163.com (X.L.); tcrz9@jnu.edu.cn (C.Z.); 2 School of Medicine, Foshan University, Foshan 528225, China

**Keywords:** diabetic wounds, copper peroxide, dual-responsive

## Abstract

Diabetic foot ulcers (DFUs) present a major clinical challenge because of persistent hyperglycemia, hypoxia, impaired angiogenesis, and chronic inflammation. Conventional dressings have limited capacity to modulate these pathological processes. Here, we report a glucose/pH dual-responsive hydrogel, denoted CBOD, constructed through a dynamic covalent network comprising Schiff base and boronate ester bonds between phenylboronic acid-grafted chitosan (CS-BA) and dopamine-modified oxidized sodium alginate (OSA-DA). Copper peroxide (CuO_2_) nanoparticles are encapsulated within this network to form CuO_2_/CBOD. Under acidic (pH 5.5) and high-glucose conditions, competitive binding and bond hydrolysis promote network relaxation, facilitating the stimuli-dependent release of oxygen and copper ions. In vitro assessments demonstrate good cytocompatibility and enhanced migration and tube formation of human umbilical vein endothelial cells (HUVECs). In a diabetic rat model of full-thickness excisional wounds, the hydrogel accelerates wound closure, with closure approaching 100% by day 14. Treatment is associated with reduced expression of the M1-associated markers interleukin-1β (IL-1β) and cluster of differentiation 80 (CD80), increased expression of the M2-associated markers interleukin-10 (IL-10) and cluster of differentiation 163 (CD163), and enhanced expression of the vascular markers cluster of differentiation 31 (CD31) and alpha-smooth muscle actin (α-SMA). These findings support the potential of CuO_2_/CBOD as a multifunctional dressing for diabetic wound management.

## 1. Introduction

The skin serves as the primary barrier against the external environment, and its structural integrity is significant to human health [1]. While wounds heal through a coordinated sequence of hemostasis, inflammation, proliferation, and remodeling, this process is disrupted in patients with diabetes [2,3]. Diabetic foot ulcers (DFUs), a serious complication of diabetes mellitus, often remain in a prolonged inflammatory phase, causing substantial morbidity and imposing a global economic burden [4,5]. The persistence of DFUs is associated with a dysregulated local microenvironment. Prolonged hyperglycemia triggers the accumulation of advanced glycation end products and severe oxidative stress, directly impairing cellular function [6]. Simultaneously, peripheral microangiopathy exacerbates tissue hypoxia. The resulting ischemic and pro-inflammatory microenvironment suppresses angiogenic responses, thereby delaying wound healing and progression beyond the inflammatory phase [7,8,9].

Conventional clinical interventions, including surgical debridement, negative pressure wound therapy [10], and moisture-retentive dressings, play important roles in wound management but may not adequately address persistent hypoxia and impaired angiogenesis [11]. Furthermore, the topical application of exogenous growth factors (e.g., vascular endothelial growth factor [VEGF] and epidermal growth factor [EGF]) is limited by rapid enzymatic degradation and high therapeutic costs. Consequently, there is a need to develop responsive biomaterials capable of sensing the diabetic microenvironment and delivering multiple therapeutic effects [12,13,14].

In recent years, stimuli-responsive hydrogels, that can react to pH [15], glucose [16], reactive oxygen species (ROS) [17], temperature [18], and other pathological cues, have emerged as a promising platform in advanced wound management. Owing to their highly hydrated, porous, and tunable three-dimensional extracellular matrix (ECM)-like networks, hydrogels provide an ideal moist environment for accelerated re-epithelialization [19,20]. Recent studies have demonstrated that hydrogel properties and polymer–cell interactions can significantly influence HUVEC migration and capillary-like network formation, including through receptor-mediated signaling pathways such as CD44 [21]. To confer environmental adaptability, natural polysaccharides such as chitosan (CS) and sodium alginate (SA) have been extensively functionalized [22,23,24]. Specifically, the incorporation of dynamic covalent bonds, such as pH-sensitive Schiff base linkages and glucose-responsive boronate esters, enables hydrogels to undergo structural transformation in response to specific pathological triggers [25,26]. The diabetic wound microenvironment is typically acidic and enriched with glucose in the exudate [15]. Thus, the development of a dual-responsive hydrogel provides a promising platform for the spatiotemporally controlled delivery of therapeutic agents.

In addition to a responsive carrier, an effective therapeutic payload is needed to alleviate hypoxia and promote neovascularization. Copper peroxide (CuO_2_) nanoparticles have attracted interest in biomedical applications [27,28]. Upon degradation in an acidic environment, CuO_2_ serves as an in situ oxygen-generating depot, with the potential to alleviate tissue hypoxia and support cellular metabolism [29,30]. The concomitant release of Cu^2+^ can upregulate angiogenic factors such as VEGF, stimulating endothelial cell migration and capillary tube formation [31,32,33]. However, the direct application of free CuO_2_ nanoparticles in vivo is limited by potentially rapid degradation, which can cause local copper accumulation, cytotoxicity, and oxidative stress [34]. Despite recent advances in carrier-mediated delivery [35,36,37,38,39], many conventional systems depend on passive diffusion or gradual matrix degradation, with limited responsiveness to the complex diabetic wound microenvironment. As a result, they often exhibit burst release, limited term efficacy, and a mismatch with the changing oxygen and copper requirements of wound healing [40,41].

To address these challenges, we designed a glucose/pH dual-responsive hydrogel (CBOD) loaded with CuO_2_ nanoparticles to combine oxygen delivery with the promotion of angiogenesis for diabetic wound healing. Phenylboronic acid-modified chitosan (CS-BA) and dopamine-modified oxidized sodium alginate (OSA-DA) were synthesized as polymer components. The CBOD hydrogel network was crosslinked via two types of dynamic covalent bonds, namely Schiff base bonds formed between the amine groups of chitosan and the aldehyde groups of oxidized alginate, and boronate ester bonds formed between phenylboronic acid and catechol groups of dopamine. CuO_2_ nanoparticles were encapsulated within this dynamic matrix. We hypothesized that after application to a diabetic wound, the acidic and high-glucose microenvironment would destabilize the hydrogel network through glucose competition and acid-induced hydrolysis of dynamic bonds. This network relaxation was expected to regulate CuO_2_ exposure and support the sustained, stimulus-dependent release of oxygen and Cu^2+^. This study systematically evaluated the structural characteristics, dual-responsive release kinetics, and in vitro pro-angiogenic activity of the CuO_2_/CBOD hydrogel, followed by in vivo evaluation using a diabetic rat full-thickness excisional wound model. This study systematically evaluated the structural characteristics, dual-responsive release kinetics, and in vitro pro-angiogenic activity of the CuO_2_/CBOD hydrogel, followed by in vivo evaluation using a diabetic rat full-thickness excisional wound model (Scheme 1).

**Scheme 1:**
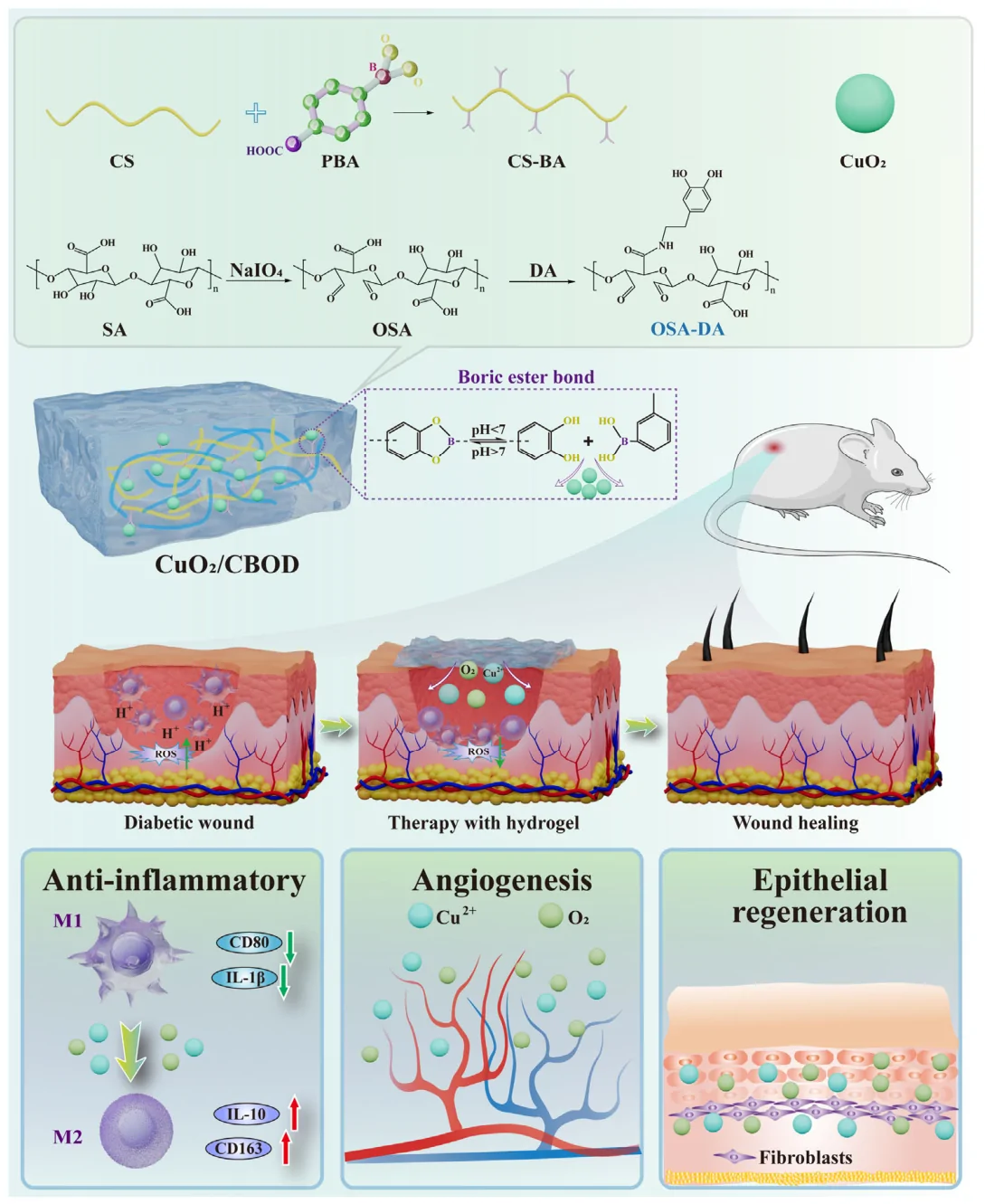
CBOD dual-responsive release of CuO_2_ in the treatment of diabetes wound healing.

## 2. Results and Discussion

### 2.1. Characterization of CuO_2_ Nanoparticles

CuO_2_ nanoparticles, the active therapeutic component, were synthesized by chemical precipitation using PVP as a stabilizer to control particle size and limit agglomeration. The morphology of the as-prepared CuO_2_ nanoparticles was examined by TEM (Figure 1A). The nanoparticles exhibited an irregular, sheet-like nanostructure with apparent stacking and aggregation. The corresponding energy-dispersive X-ray spectroscopy (EDS) elemental maps revealed that Cu and O were uniformly distributed and well colocalized throughout the nanostructures. Dynamic light scattering (DLS) analysis demonstrated a narrow size distribution ranging from 200 to 350 nm (Figure 1B). The chemical composition and oxidation states of the nanoparticles were verified using XPS. The high-resolution Cu 2p spectrum exhibited two prominent peaks at 933.5 and 953.5 eV, accompanied by satellite peaks at 942.1 and 962.1 eV, confirming the presence of copper (II). The O 1s spectrum displayed peaks at 530.7 and 532.5 eV, assigned to C=O and O-O peroxide bonds, respectively, supporting the formation of a peroxide structure and the presence of PVP (Figure 1C–E).

### 2.2. Characterization of Hydrogel Precursors

To prepare the responsive matrix, natural polysaccharides were chemically modified. Chitosan was conjugated with PBA via amide coupling to yield CS-BA. FTIR spectroscopy of CS-BA revealed characteristic absorption peaks of the benzene ring at 795 cm^−1^ for out-of-plane bending and 1540 cm^−1^ for skeletal vibration (Figure 2A). ^1^H NMR further supported PBA grafting, with distinct aromatic proton signals appearing between 7.4 and 8.2 ppm (Figure 2B). Quantitative analysis of the NMR integrals relative to the chitosan backbone signals yielded a PBA grafting degree of 28.5 ± 0.5%. Concurrently, DA was grafted onto oxidized sodium alginate (OSA) to form OSA-DA. The FTIR spectrum showed a new peak at 1734 cm^−1^, corresponding to the C=O stretching of the aldehyde groups introduced by oxidation, along with peaks at 1530 and 1285 cm^−1^ associated with the DA aromatic ring and phenolic hydroxyl groups (Figure 2C). The ^1^H NMR spectrum of OSA-DA exhibited characteristic aromatic proton signals of DA at 6.89, 6.84, and 6.75 ppm, supporting DA grafting onto OSA (Figure 2D). Potentiometric titration yielded an OSA oxidation degree of 35.2 ± 1.9%. Quantitative analysis of the NMR integrals relative to the OSA backbone signals yielded a DA grafting degree of 27.5 ± 2.1%.

### 2.3. Microstructure and Rheological Properties of the Hydrogels

The dual-responsive hydrogel (CBOD) was fabricated by mixing CS-BA and OSA-DA, leading to the spontaneous formation of Schiff base bonds between amines and aldehydes as well as boronate ester bonds between PBA and catechol groups. SEM images of the lyophilized hydrogels revealed a three-dimensional interconnected porous structure with pore sizes ranging from 100 to 200 μm (Figure S1). The incorporation of CuO_2_ nanoparticles did not visibly disrupt this porous structure. This porous structure may facilitate exudation and oxygen transport.

Rheological analyses were performed to evaluate the viscoelastic behavior of the hydrogels. Frequency sweep tests demonstrated that the storage modulus (G′) consistently exceeded the loss modulus (G″) across the 0.1–100 rad/s range, indicating predominantly elastic, solid-like behavior under the tested conditions (Figure 2E). The incorporation of CuO_2_ increased both G′ and G″, possibly because the nanoparticles provided additional physical crosslinking sites and increased the effective network density. Strain sweep measurements revealed that the CBOD hydrogel exhibited a critical strain of approximately 700%, whereas the CuO_2_/CBOD hydrogel exhibited network yielding at about 250% strain, at which G′ and G″ crossed. This decrease in the yielding strain may reflect stress concentration around rigid CuO_2_ nanoparticles at large deformations (Figure 2F).

Both hydrogels rapidly absorbed water and reached swelling equilibrium within 12 h (Figure 2G). The equilibrium swelling ratio of CuO_2_/CBOD was slightly lower than that of CBOD, consistent with its denser, nanoparticle-reinforced network. The fluid uptake of both hydrogels suggests their potential for wound exudate management. In addition, adhesion tests on porcine skin and bending tests on finger surfaces further demonstrated the tissue adhesion of the CuO_2_/CBOD hydrogel (Videos S1 and S2; Figure S2).

### 2.4. Release Kinetics of Cu^2+^ and Oxygen

To evaluate the stimulus responsiveness of the CuO_2_/CBOD hydrogel, the release kinetics of Cu^2+^ were monitored using ICP-MS at different pH values and glucose concentrations. The hydrogel exhibited minimal Cu^2+^ release in glucose-free buffer at pH 7.4 (0 mg/mL glucose). However, when exposed to acidic, high-glucose conditions (pH 5.5, 5 mg/mL glucose), Cu^2+^ release accelerated, reaching approximately 48% cumulative release at 72 h. This responsive release is consistent with glucose-induced disruption of boronate ester bonds and acid-promoted hydrolysis of Schiff base bonds. These changes may loosen the hydrogel network and increase the exposure of CuO_2_ nanoparticles to the acidic medium, thereby facilitating their decomposition (Figure 2H).

The capacity of the hydrogel to generate oxygen in situ was assessed. Compared with CBOD, the CuO_2_/CBOD hydrogel exhibited sustained oxygen release, with the measured profile reaching a plateau around days 4–5. Oxygen generation increased significantly under acidic conditions and was further enhanced in the presence of high glucose. This sustained oxygen release may provide a prolonged oxygen supply to alleviate wound hypoxia and support tissue repair (Figure 2I).

In the glucose-free buffer (pH 7.4, 0 mg/mL glucose), the hydrogel remained largely intact, retaining approximately 92% of its initial mass after 12 days. Degradation was pH-dependent: acidification to pH 5.5 accelerated degradation, reducing the remaining mass to approximately 55%. The presence of glucose also accelerated degradation at physiological pH, consistent with glucose-triggered disruption of the boronate ester bonds. At pH 5.5, the addition of glucose resulted in a slightly higher remaining mass than in the glucose-free acidic medium, suggesting that acid-driven network degradation may predominate under these conditions, whereas the contribution of glucose-responsive boronate ester bonds is more apparent at pH 7.4. Together, these results demonstrate pH- and glucose-dependent changes in hydrogel degradation, supporting the dual-responsive design (Figure S3).

### 2.5. Cytocompatibility of CuO_2_/CBOD Hydrogels

The cytotoxicity of free CuO_2_ nanoparticles was evaluated in L929 fibroblasts. Cell viability varied with CuO_2_ concentration. Concentrations below 50 μg/mL maintained viability above 90%, whereas concentrations exceeding 100 μg/mL induced significant cytotoxicity, possibly owing to oxidative stress induced by excessive Cu^2+^ and H_2_O_2_ (Figure 3A,B). These results guided the selection of the CuO_2_ loading for the hydrogel. To assess the cellular oxidative response, intracellular ROS levels were quantified using fluorescence staining and flow cytometry (Figure 3E,F and Figure S4). The CuO_2_-loaded hydrogel produced only a slight increase in ROS levels above baseline, whereas the H_2_O_2_ positive control induced a pronounced increase. Furthermore, live/dead staining and CCK-8 assays supported the cytocompatibility of the hydrogel. By day 5, HUVECs co-cultured with the CuO_2_/CBOD hydrogel exhibited proliferation and maintained multipolar morphologies, whereas control cells showed morphological changes suggestive of senescence (Figure 3C,D).

### 2.6. Pro-Angiogenic Effects of CuO_2_/CBOD Hydrogels

Impaired vascularization is a major barrier to diabetic tissue repair. The pro-angiogenic capacity of the hydrogel was evaluated using HUVECs. The scratch assay showed that CuO_2_ enhanced HUVEC migration, with the greatest effect at 50 μg/mL. At 72 h, the relative migration rate at this concentration was 3.81 times that of the control. At 100 μg/mL, this effect was reduced, highlighting the importance of controlling CuO_2_ (Figure 4A,C). The tube formation assay provided further evidence of pro-angiogenic activity. HUVECs treated with the CuO_2_/CBOD hydrogel extract formed interconnected capillary-like networks within 12 h, yielding 278 tube junctions, more than those observed with the CBOD hydrogel or the control (Figure 4B,D). This pro-angiogenic response may be associated with the sustained release of Cu^2+^ [42,43].

### 2.7. Diabetic Wound Healing In Vivo

The therapeutic efficacy of the CuO_2_/CBOD hydrogel was evaluated as a full-thickness cutaneous wound model in STZ-induced diabetic rats. Macroscopic wound closure was monitored over 14 days (Figure 5A,B). By day 3, the CuO_2_/CBOD group exhibited a significantly higher wound closure rate of 61.18% compared to 38.94% for the 3M commercial dressing and 37.09% for the untreated control, indicating accelerated early wound closure. By day 14, the wounds treated with CuO_2_/CBOD achieved near-complete closure (99.88%) and showed improved macroscopic tissue appearance, whereas the control group exhibited 87.63% closure (Figure 5C).

H&E and Masson’s trichrome staining were performed to assess tissue structure and evaluate the healing process. On day 3, extensive inflammatory cell infiltration was observed in the control group, whereas the CuO_2_/CBOD group exhibited a substantially reduced inflammatory response and early signs of granulation tissue formation. By day 14, H&E staining revealed that the CuO_2_/CBOD-treated wounds displayed a continuous, stratified epithelial layer, closely resembling healthy skin. Masson’s trichrome staining further demonstrated that the CuO_2_/CBOD group exhibited denser, more organized collagen bundles than those in the control and 3M commercial dressing groups (Figure 5D,E).

### 2.8. Modulation of the Inflammatory In Vivo

Chronic diabetic wounds often persist in a pro-inflammatory state that hinders the transition to the proliferative phase. To assess the immunomodulatory effects of the hydrogels, IHC staining was performed to examine pro-inflammatory/M1-associated markers (IL-1β and CD80; Figure 6A–C) and anti-inflammatory/M2-associated markers (IL-10 and CD163; Figure 6D–F). IHC staining showed that treatment with CuO_2_/CBOD significantly reduced the expression of the pro-inflammatory cytokine IL-1β and the M1 macrophage marker CD80, consistent with reduced local inflammation. Concurrently, CuO_2_/CBOD increased the expression of the anti-inflammatory cytokine IL-10 and the M2 macrophage marker CD163, consistent with a shift toward an anti-inflammatory, M2-associated phenotype that may support tissue repair. Collectively, these findings suggest that CuO_2_/CBOD alleviates early wound inflammation and modulates the local immune microenvironment, which may support subsequent angiogenesis, extracellular matrix remodeling, and tissue repair.

### 2.9. Modulation of the Angiogenic In Vivo

In vivo angiogenesis was assessed using immunofluorescence staining for CD31 (an endothelial cell marker) and α-SMA (a smooth muscle-associated marker) (Figure 7A,B). On days 7 and 14, the CuO_2_/CBOD group exhibited the highest fluorescence intensity for CD31, consistent with enhanced neovascularization. The concomitant increase in α-SMA staining may be consistent with increased mural cell coverage and vessel maturation (Figure 7C,D). These findings suggest that the release of Cu^2+^ and oxygen may support vascular repair in diabetic wound tissue.

## 3. Conclusions

We developed a glucose/pH dual-responsive hydrogel (CuO_2_/CBOD) to address hypoxia, impaired angiogenesis, and inflammation in diabetic wounds. By incorporating dynamic Schiff base and boronate ester bonds, the material exhibits responsive release in acidic and high-glucose microenvironments. This stimulus responsiveness regulates CuO_2_ decomposition and supports the sustained release of oxygen and Cu^2+^. In vitro and in vivo findings demonstrate that this delivery system promotes angiogenesis and reduces local inflammation. The CuO_2_/CBOD dressing promoted tissue repair and achieved near-complete closure of diabetic wounds within 14 days. These findings support further investigation of CuO_2_/CBOD as a responsive delivery platform for diabetic wound management.

## 4. Materials and Methods

### 4.1. Materials

Anhydrous copper (II) chloride (CuCl_2_), polyvinylpyrrolidone (PVP, 24 kDa), 30% hydrogen peroxide (H_2_O_2_), chitosan (CS, Mw = 100 kDa), and sodium periodate (NaIO_4_) were purchased from Macklin. Sodium alginate (SA), 3-carboxyphenylboronic acid (PBA), dopamine hydrochloride (DA), N-hydroxy succinimide (NHS), and 1-ethyl-3-(3-dimethylaminopropyl) carbodiimide hydrochloride (EDC) were purchased from Sigma. The Cell Counting Kit-8 (CCK-8) assay kit was purchased from DOJINDO. Dulbecco’s modified Eagle’s medium (DMEM), fetal bovine serum (FBS), and phosphate-buffered saline (PBS) were purchased from Gibco (Carlsbad, CA, USA).

### 4.2. Preparation and Characterization of CuO_2_ Nanoparticles

CuO_2_ nanoparticles were synthesized via a modified precipitation method. Briefly, 5 g of PVP was dissolved in 50 mL of CuCl_2_ solution (0.01 mol/L). After vigorous mixing, 50 mL of NaOH solution (0.02 mol/L) was added, and the mixture was magnetically stirred for 10 min. Subsequently, 1 mL of 30% H_2_O_2_ was added dropwise. The mixture was stirred for 30 min, and the resulting precipitate was collected by centrifugation, washed three times with deionized water, and vacuum-dried to obtain CuO_2_ nanoparticles. The hydrodynamic size of the CuO_2_ nanoparticles was measured using a Zetasizer-ZS90 (Malvern Instruments, Malvern, UK). The morphology of the CuO_2_ nanoparticles was observed using transmission electron microscopy (TEM, Tecnai 12, Philips, Amsterdam, The Netherlands). The elemental composition and oxidation states were analyzed using X-ray photoelectron spectroscopy (XPS, Thermo Kalpha, Waltham, MA, USA).

### 4.3. Synthesis of CS-BA and OSA-DA Polymers

To synthesize CS-BA, 0.5 g of CS was dissolved in 400 mL of 0.3 wt% acetic acid. Separately, 2.05 g of PBA was dissolved in 50 mL of methanol and activated with EDC/NHS for 30 min. The activated PBA was added to the CS solution, stirred at room temperature for 24 h, dialyzed against deionized water for 3 days, and lyophilized. For OSA-DA, 5 g of SA was oxidized using NaIO_4_ (1:1 molar ratio) in the dark for 6 h. The reaction was quenched with ethylene glycol. The resulting OSA (3.6 g) was dissolved in water, activated with EDC/NHS, and reacted with 2.84 g of DA under a nitrogen atmosphere for 12 h to prevent premature dopamine oxidation. The product was dialyzed and lyophilized to yield OSA-DA. The chemical structures of CS, PBA, SA, DA, CS-BA, and OSA-DA were characterized using Fourier-transform infrared spectroscopy (FTIR; Spectrum Two, PerkinElmer, Hopkinton, MA, USA) and proton nuclear magnetic resonance spectroscopy (1H NMR; AVANCE III 600, Bruker, Billerica, MA, USA). D_2_O was used as the solvent for ^1^H NMR measurements.

### 4.4. Preparation of the CuO_2_/CBOD Hydrogels

The CBOD hydrogel was prepared by mixing a 2 wt% CS-BA solution with a 6 wt% OSA-DA solution at a volume ratio of 3:1, followed by the addition of 0.1 M NaOH to facilitate rapid crosslinking. For the composite hydrogel, 1 mg of CuO_2_ was dispersed in 10 mL of deionized water by sonication before the respective polymers were dissolved. The same crosslinking procedure was used, yielding the CuO_2_/CBOD hydrogel. The morphology of the hydrogels was examined by scanning electron microscopy (SEM; SU1000, Hitachi, Tokyo, Japan). Viscoelastic properties were evaluated at 25 °C using a rotational rheometer (TA Instruments, New Castle, DE, USA). Frequency sweeps (0.1–100 rad/s, constant strain of 0.05%) and strain sweeps (1–1000%, constant angular frequency of 25 rad/s) were performed to assess the viscoelastic response of the hydrogels.

### 4.5. In Vitro Release and Degradation

To evaluate the oxygen-generating capacity of CuO_2_/CBOD under simulated wound conditions, standardized cylindrical hydrogel samples (Ø 10 mm × 3 mm) were immersed in 10 mL of PBS under four pH/glucose conditions: (i) pH 7.4, 0 mg/mL glucose; (ii) pH 5.5, 0 mg/mL glucose; (iii) pH 7.4, 5 mg/mL glucose; and (iv) pH 5.5, 5 mg/mL glucose. Prior to use, the PBS was deoxygenated by purging with nitrogen (N_2_). Each hydrogel sample was placed in a vial containing the release medium. The medium was purged with N_2_ for 1 min to remove residual dissolved oxygen, and the vial was immediately sealed with a gas-tight cap. The vials were incubated at 37 °C with shaking (150 rpm). Dissolved oxygen (DO) concentrations were monitored at predetermined time intervals using a portable DO meter (JPB-607A, INESA, Shanghai, China). At each sampling time point, the release medium was collected and replaced with an equal volume of PBS; the vial was then purged again with N_2_ for 1 min and resealed. CBOD hydrogels processed under identical conditions served as controls. Oxygen generation was reported as the net increase in DO concentration relative to the corresponding CBOD control. Cu^2+^ concentrations in the collected media were quantified by inductively coupled plasma mass spectrometry (ICP-MS) under the same conditions, and cumulative release curves were plotted.

The degradation behavior of the CuO_2_/CBOD hydrogel was evaluated gravimetrically under four pH/glucose conditions: (i) pH 7.4, 0 mg/mL glucose; (ii) pH 5.5, 0 mg/mL glucose; (iii) pH 7.4, 5 mg/mL glucose; and (iv) pH 5.5, 5 mg/mL glucose. Standardized cylindrical hydrogel samples (Ø 10 mm × 3 mm) were prepared, frozen at −80 °C, and lyophilized to obtain the initial dry weight (W_0_). Each sample was then immersed in 10 mL of the corresponding PBS in a sealed vial and incubated at 37 °C with shaking (150 rpm). The release medium was refreshed every 2 days to maintain the specified pH and glucose concentration. At predetermined time intervals (0, 2, 4, and 12 h, and during days 1–12), samples were removed, lyophilized, and weighed to obtain the dry weight at time t (W_t_). The percentage of the remaining mass was calculated according to the following equation:

Remaining mass (%) = (W_t_/W_0_) × 100%

All measurements were performed in triplicate, and the results are expressed as the mean ± standard deviation (SD).

### 4.6. In Vitro Cell Studies

L929 fibroblasts and human umbilical vein endothelial cells (HUVECs, CL-0675) were purchased from Wuhan Procell Company (Wuhan, China) and cultured using standard cell culture protocols. Cell viability was assessed using CCK-8 and live/dead staining kits. Intracellular ROS levels were measured using a 2′,7′-dichlorodihydrofluorescein diacetate (DCFH-DA) probe and flow cytometry. Detailed methods are provided in the Supporting Information.

### 4.7. In Vitro Pro-Angiogenic Activity

#### 4.7.1. HUVECs Migration Assay

The effects of CuO_2_ on cell migration were assessed using a scratch wound-healing assay. Confluent cell monolayers in 6-well plates were scratched with a sterile pipette tip, washed with PBS, and treated with low-serum medium (containing 2% FBS) supplemented with CuO_2_ at concentrations of 0, 25, 50, or 100 µg/mL. HUVECs migration rates were calculated at 0, 12, 24, 48, and 72 h.

#### 4.7.2. Tube Formation Assay

In vitro angiogenic potential was further evaluated using a tube formation assay. Precooled 96-well plates were coated with Matrigel (100 μL/well) and incubated at 37 °C for polymerization. HUVECs (1 × 10^4^ cells/well) pretreated with extracts (Control, 50 μg/mL CuO_2_, CBOD, and CuO_2_/CBOD) were seeded onto the Matrigel. After 6 and 12 h of incubation, capillary-like network formation was examined under a microscope. The number of tube junctions was quantified using ImageJ (Image 1.53t).

### 4.8. In Vivo Evaluation in a Diabetic Wound Model

#### 4.8.1. Establishment of the Diabetic Rat Wound Model and Treatment Groups

Female Sprague-Dawley (SD) rats aged 6–8 weeks were used in this study. All animal procedures were approved by the Animal Ethics Committee of Ruige Biotechnology, China (Approval No. IACUC 20240109–001). Diabetes was induced by a single intraperitoneal injection of streptozotocin (STZ, 55 mg/kg). Rats with stable fasting blood glucose levels ≥ 16.7 mmol/L were included. Under anesthesia, a single full-thickness circular wound (10 mm in diameter) was created on the dorsum of each rat. Each animal represented an independent experimental unit. Rats were randomly assigned to four groups (n = 9 rats per group): Control (no treatment), 3M (commercial dressing), CBOD, and CuO_2_/CBOD hydrogel. Dressings were replaced every 3 days. Macroscopic images of the wounds were acquired on days 0, 3, 7, and 14, and wound areas were measured using ImageJ to calculate wound closure.

#### 4.8.2. Histological Analysis

Rats were euthanized on days 3, 7, and 14 after surgery (n = 3 rats per group at each time point), and wound tissues were collected for histological and immunohistochemical analyses of healing. At each designated time point, wound tissues with a 5 mm margin of surrounding skin were excised, fixed in 4% paraformaldehyde, paraffin-embedded, and sectioned at a thickness of 4 μm. Hematoxylin and eosin (H&E) staining was performed at all three time points to assess granulation tissue formation and inflammatory cell infiltration, while Masson’s trichrome staining (days 3, 7 and 14) was used to evaluate collagen deposition and remodeling.

#### 4.8.3. Immunohistochemical Staining

For immunohistochemical analysis, paraffin-embedded sections were deparaffinized and rehydrated, followed by antigen retrieval. The sections were then blocked to prevent nonspecific antibody binding and incubated with primary and secondary antibodies. Target staining was then visualized and quantified in the tissue sections.

To characterize the inflammatory microenvironment, immunohistochemical (IHC) staining was performed for pro-inflammatory/M1-associated markers (IL-1β and CD80) and anti-inflammatory/M2-associated markers (IL-10 and CD163) to assess changes in inflammation and macrophage-associated phenotypes. Immunofluorescence (IF) staining for CD31 and α-SMA was conducted to assess neovascularization within the wound.

### 4.9. Statistical Analysis

All quantitative data are presented as the mean ± standard deviation (SD), and error bars in the figures represent SD. Unless otherwise stated, experiments were performed with at least three independent replicates, and n denotes the number of independent samples or animals per group. Data was analyzed using Origin 2022 software (OriginPro 2021, OriginLab, Northampton, MA, USA). Image-based quantification, including wound area, tube junctions, and the positive area or fluorescence intensity of immunohistochemical and immunofluorescence staining, was performed using ImageJ (Image 1.53t, NIH, Stapleton, NY, USA) on randomly selected fields from at least three sections per animal; values from multiple fields of the same animal were averaged to yield a single value per animal.

Normality of the data was assessed using the Shapiro–Wilk test, and homogeneity of variance was assessed using Levene’s test. Comparisons between two groups were performed using an unpaired two-tailed Student’s *t*-test when the data were normally distributed with equal variances. Comparisons among three or more groups were performed using one-way analysis of variance (ANOVA) followed by Tukey’s honestly significant difference (HSD) post hoc test.

In all cases, a *p* value < 0.05 was considered statistically significant. Significance levels are indicated as *p* < 0.05, *p* < 0.01, and * *p* < 0.001; ns denotes not significant.

## Figures and Tables

**Figure 1:**
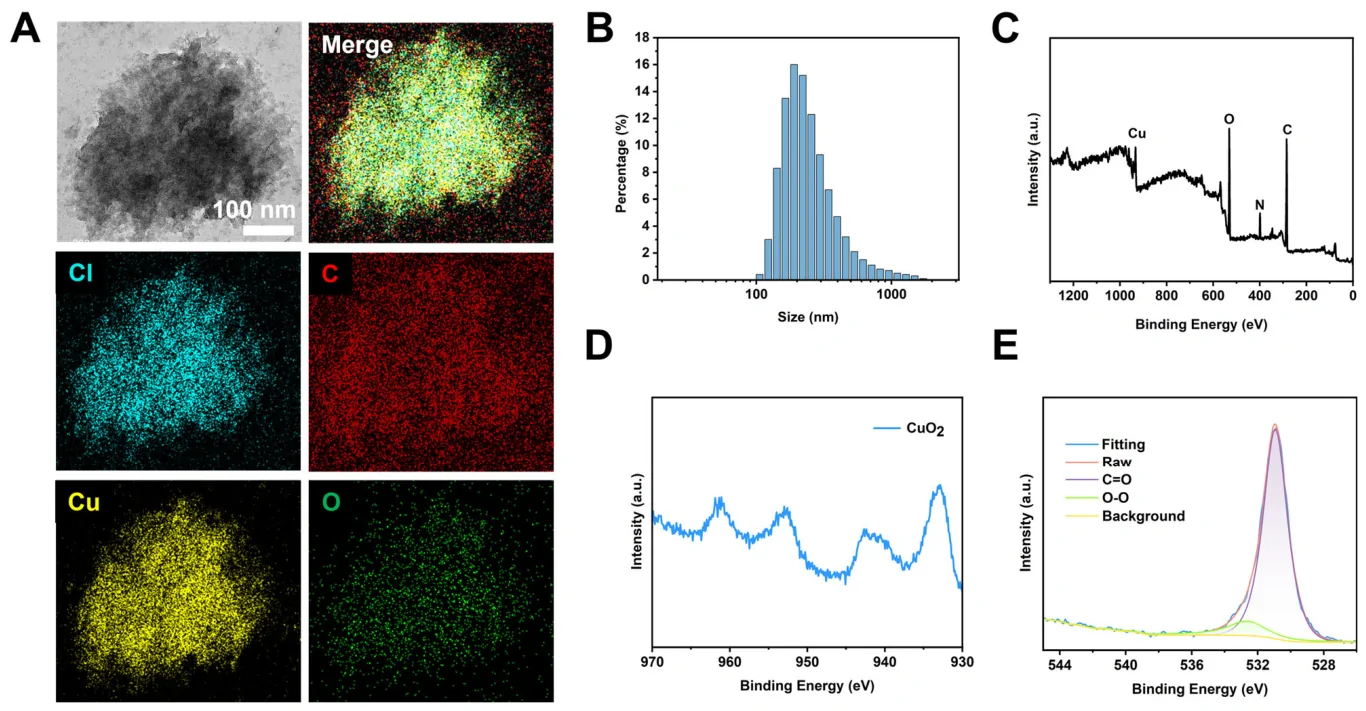
Characterization of nanoparticles. (**A**) TEM image and (**B**) size distribution of CuO_2_ nanoparticles; (**C**–**E**) High-resolution XPS spectra of CuO_2_. Scale bar = 100 nm.

**Figure 2:**
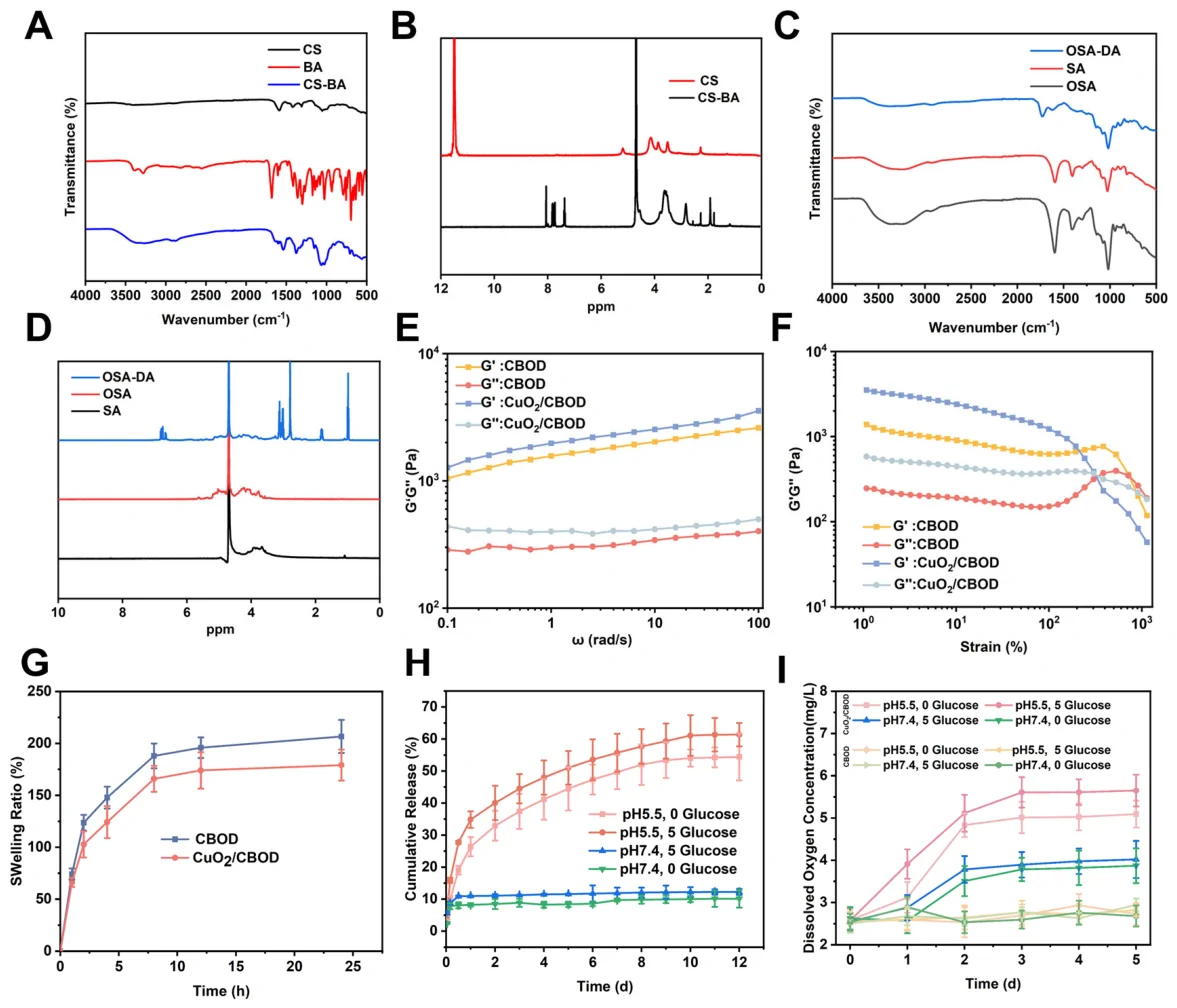
Characterization of hydrogels. (**A**) FTIR and (**B**) ^1^H NMR spectra of CS-BA; (**C**) FTIR and (**D**) ^1^H NMR spectra of OSA-DA; Rheological evaluation demonstrating (**E**) frequency sweep and (**F**) strain sweep profiles; (**G**) Swelling ratio kinetics of the hydrogels in PBS (pH 7.4) at 37 °C; (**H**) Cumulative Cu^2+^ release and (**I**) continuous O_2_ release from the CuO_2_/CBOD and CBOD hydrogel under varying pH values and glucose concentrations (n = 3).

**Figure 3:**
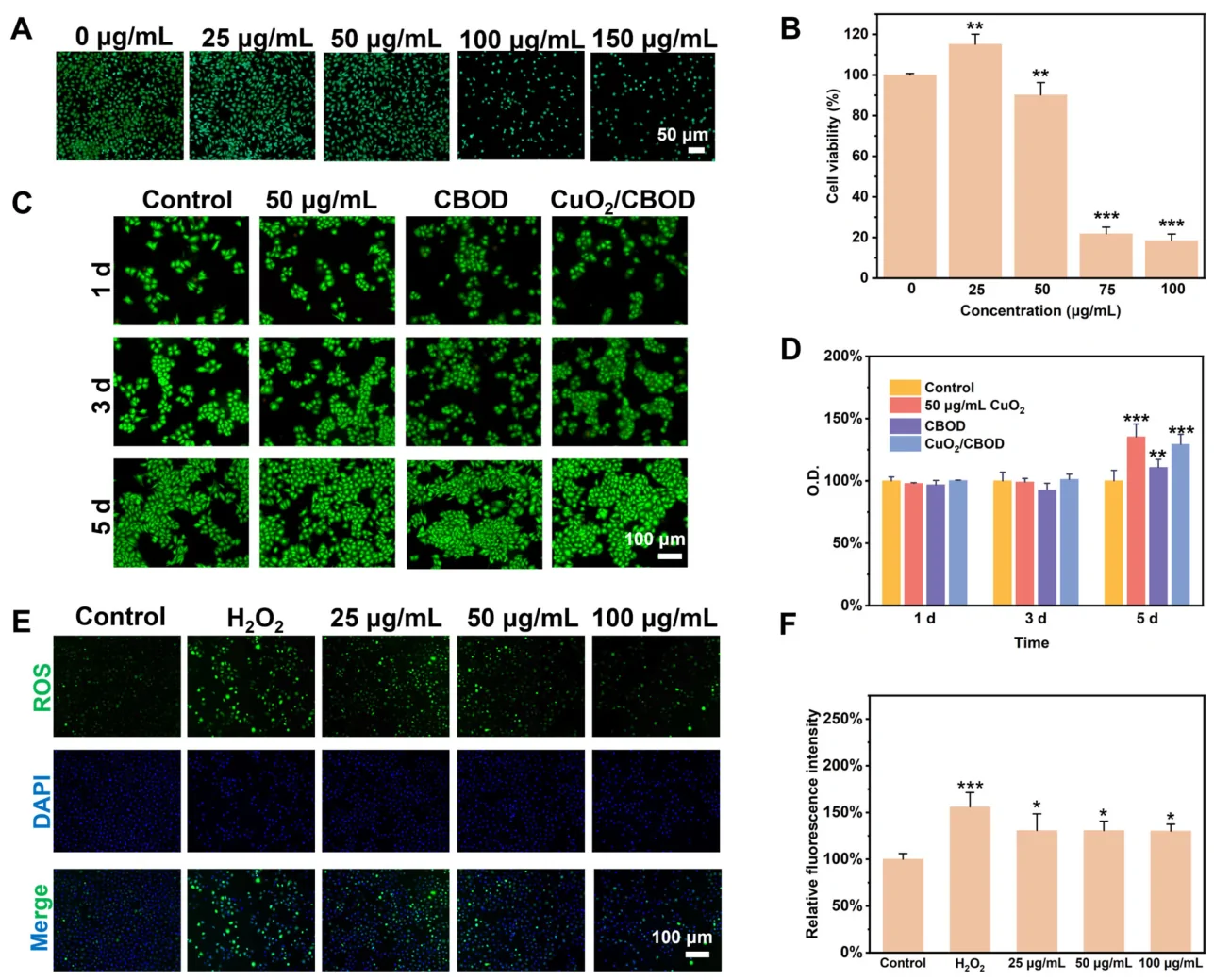
In vitro cytocompatibility. (**A**) Fluorescence microscopy images of live/dead staining and (**B**) CCK-8 proliferation assay of L929 cultured with different concentration of CuO_2_ nanoparticles. Scale bar = 50 μm. (**C**) Live/dead fluorescence staining and (**D**) CCK-8 proliferation assay of HUVECs cultured with different hydrogel extracts over 5 days. Scale bar = 100 μm; (**E**) fluorescence microscopy images of HUVECs stained with DCFH-DA. Scale bar = 100 μm. (**F**) Relative fluorescence intensity of ROS. Data are presented as mean ± SD (n = 3) (* *p* < 0.05, ** *p* < 0.01, *** *p* < 0.001).

**Figure 4:**
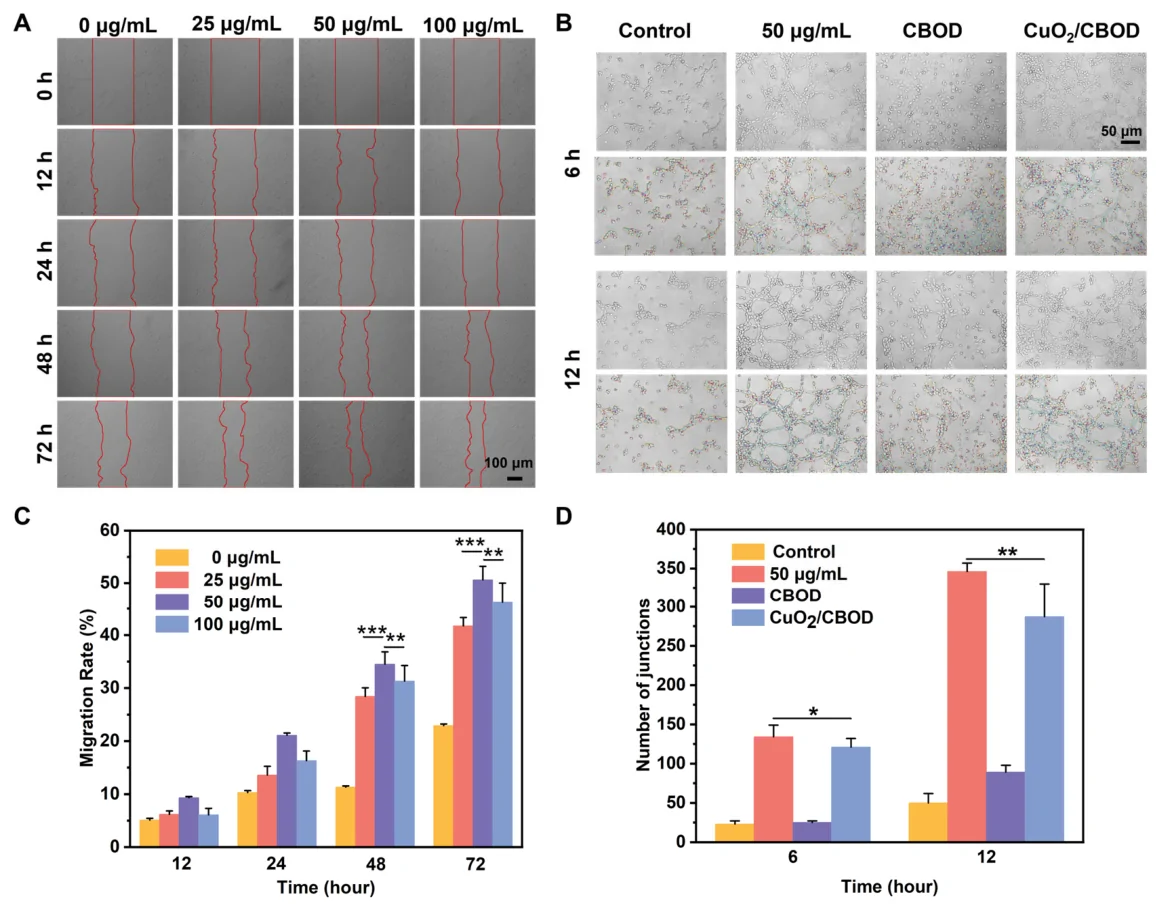
Modulation of cellular behavior and pro-angiogenic effects of CuO_2_/CBOD hydrogel. (**A**) Representative images and (**C**) quantitative analysis of HUVECs migration over 72 h. (**B**) Representative images and (**D**) quantitative analysis of endothelial tube formation (n = 3) (* *p* < 0.05, ** *p* < 0.01, *** *p* < 0.001).

**Figure 5:**
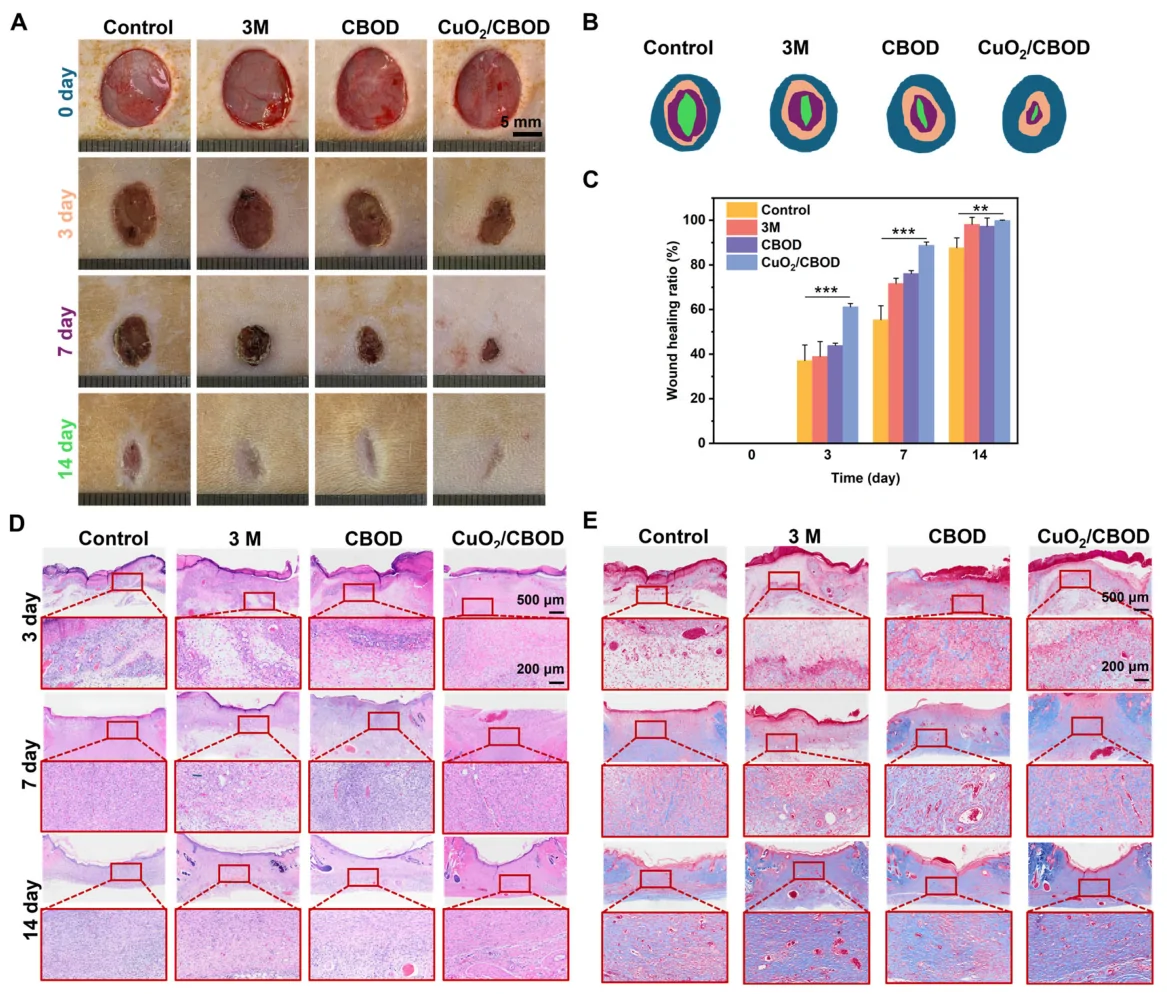
In vivo evaluation of diabetic wound healing. (**A**) Representative macroscopic images of diabetic wounds treated with different modalities at days 0, 3, 7, and 14. (**B**) Schematic illustration of wound closure. (**C**) Quantification of wound closure rates over time. (**D**) H&E staining and (**E**) Masson’s trichrome staining of the regenerated wound tissue at days 3, 7, 14 (Scale bars: 500 μm for low magnification, 200 μm for high magnification) (n = 3) (** *p* < 0.01, *** *p* < 0.001).

**Figure 6:**
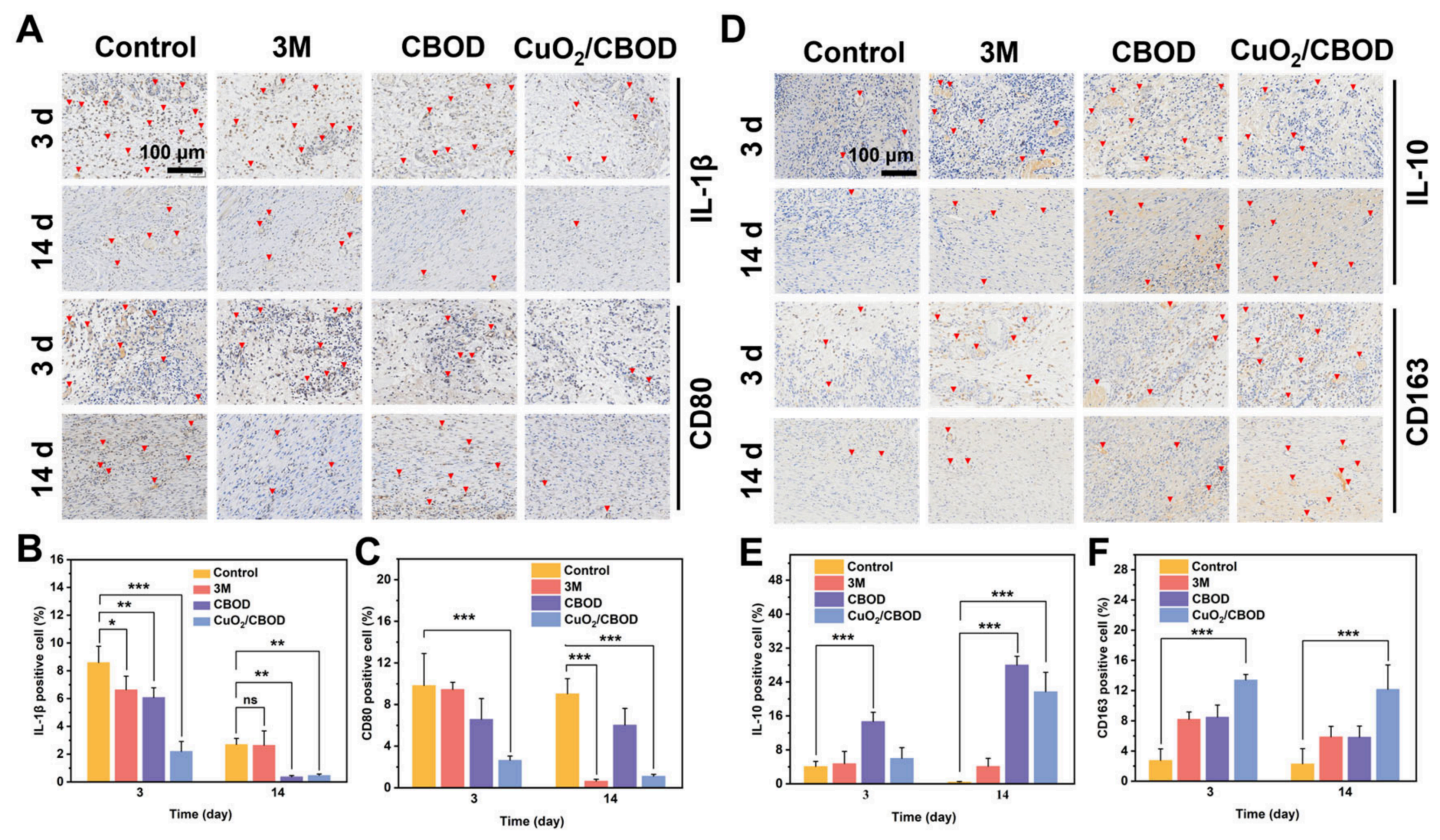
Immunohistochemical analysis of inflammatory markers in wound tissue. (**A**) Representative IHC staining images for IL-1β and CD80 at days 3 and 14. Quantitative analysis of (**B**) IL-1β and (**C**) CD80 expression. (**D**) Representative IHC staining images for IL-10 and CD163 at days 3 and 14; quantitative analysis of (**E**) IL-10 and (**F**) CD163 expression. Scale bar = 100 μm. Data are presented as mean ± SD (n = 3) (ns denotes not significant, * *p* < 0.05, ** *p* < 0.01, *** *p* < 0.001).

**Figure 7:**
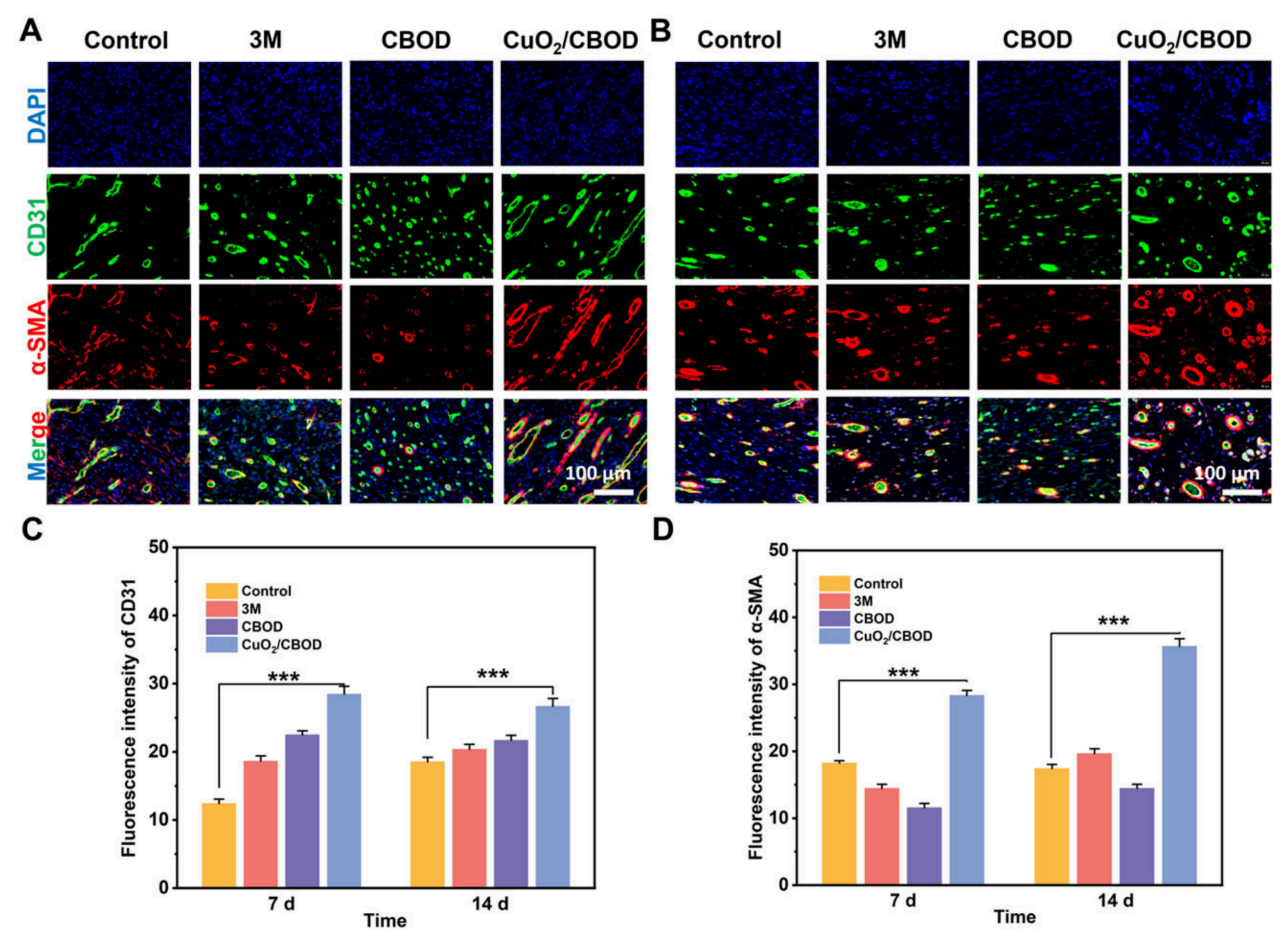
Immunofluorescence analysis of angiogenesis in vivo. Representative immunofluorescence staining images for CD31 (green) and α-SMA (red) in wound tissue sections at day 7 (**A**) and 14 (**B**). Quantitative analysis of CD31 (**C**) and α-SMA (**D**) fluorescence intensity. Data are presented as mean ± SD (n = 3) (*** *p* < 0.001).

## Data Availability

The data that support the findings of this study are available from the corresponding author upon reasonable request. All relevant data generated or analyzed during this study are included in this published article and its supplementary information files.

## Supplementary Materials

The following supporting information can be downloaded at: https://www.mdpi.com/article/10.3390/gels12100864/s1, Figure S1: The SEM images of (A,B) CBOD, (C,D) CuO_2_/CBOD freeze dried hydrogel samples; Figure S2: Hydrogel adhesion on a bent finger; Figure S3: Degradation assay of CuO_2_/CBOD; Figure S4: Representative flow cytometry histograms of DCF fluorescence intensityafter 2 h of treatment with (A) Control, (B) 0.1mM H_2_O_2_, (C) 25 μg/mL CuO_2_, (D) 50 μg/mL CuO_2_, (E) 100 μg/mL CuO_2_; (F) Quantitative analysis of mean fluorescence intensity. Data are presented as mean ± SD (n = 3) (* *p* < 0.05, ** *p* < 0.01, *** *p* < 0.001*); Video S1: Adhesion test of CuO_2_/CBOD gel on wet pigskin surface; Video S2: Adhesion test of CuO_2_/CBOD gel in water flow.
