# Supplementary material for: Copper Peroxide-Loaded Dual-Responsive Hydrogel for Enhanced Diabetic Wound Healing Through Oxygen Release and Promotion of Angiogenesis
Source: Gels. 2026 Sep 24;12(10):864. doi: 10.3390/gels12100864 (PMC13704670; doi:10.3390/gels12100864)
Supplement: Supplementary file 1 [file gels-12-00864-s001.zip › gels-4544000-supplementary.pdf]

Supporting Information

Copper Peroxide-Loaded Dual-Responsive Hydrogel for Enhanced Diabetic Wound Healing Through Oxygen Release and Promotion of Angiogenesis

Lanqin Yu [1,†], Xiang Jia [1,†], Lejia Xu [1], Xiaojun Li [1], Na Li [2,*], Lihua Li [1,*], Yifan Zhang [1,*] and Changren Zhou [1]

[1] College of Chemistry and Materials Science, Engineering Research Center of Artificial Organs and Materials, Jinan University, Guangzhou 510632, China; ylq06060604@163.com (L.Y.); jx18754297280@163.com (X.J.); m13173025825@163.com (L.X.); xj13592809047@163.com (X.L.); tcrz9@jnu.edu.cn (C.Z.)

[2] School of Medicine, Foshan University, Foshan 528225, China

* Correspondence: lina1102@fosu.edu.cn (N.L.); tlihuali@jnu.edu.cn (L.L.); yifanzhang0916@163.com (Y.Z.)

† These authors contributed equally to this work.

2.6 *In vitro* biocompatibility and ROS regulation

Hydrogel extracts were prepared by incubating CBOD and CuO_2/CBOD in cell culture medium at 37 °C for 24 h. To assess cytotoxicity, HUVECs were seeded in 24-well plates (1×10^4 cells/well) and cultured for 24 h. The medium was replaced with fresh medium containing the following interventions: Control, CBOD, CuO_2/CBOD, or 50 μg/mL CuO_2. After 24 h of co-incubation, cell viability was quantified by CCK-8 assay at 450 nm, and live/dead status was visualized by AO/EB staining under an inverted fluorescence microscope. To evaluate the capacity of the materials to regulate intracellular ROS levels, HUVECs were pre-treated with H_2O_2 to induce oxidative stress, followed by incubation with the respective sample extracts. Intracellular ROS was subsequently labeled with DCFH-DA probe and assessed by fluorescence microscopy and flow cytometry. Additionally, the cytotoxicity of CuO_2 nanoparticles across a broader concentration range (25, 50, 100, and 150 μg/mL) was independently evaluated by CCK-8 assay and AO/EB staining using L-929 cells, following the same protocol described above.

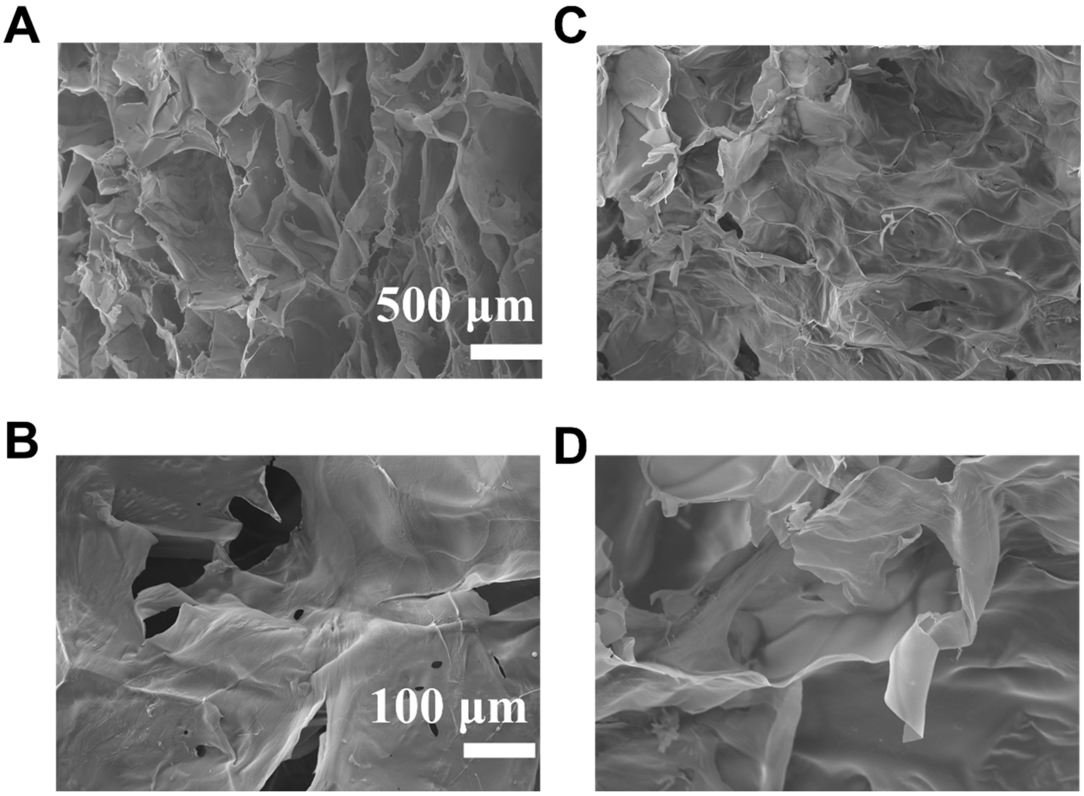

Figure S1. The SEM images of (A- B) CBOD, (C- D) CuO_2/CBOD freeze dried hydrogel samples.

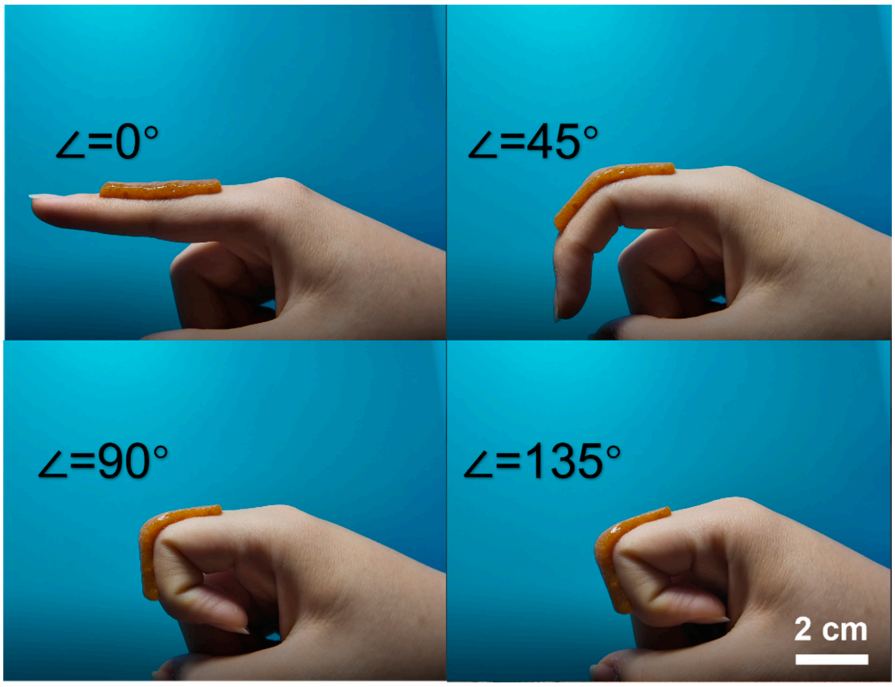

Figure S2. Hydrogel adhesion on a bent finger.

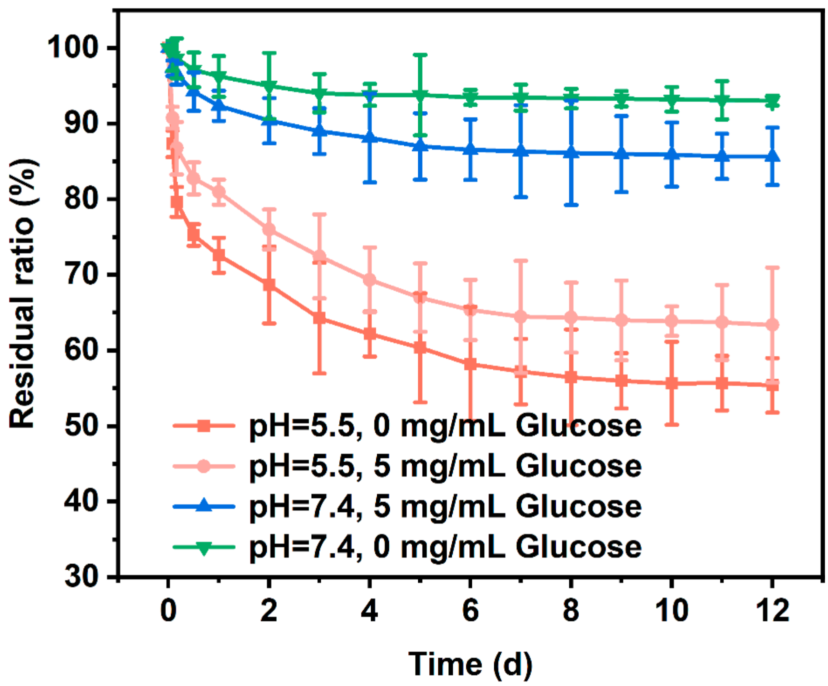

Figure S3. Degradation assay of CuO_2/CBOD.

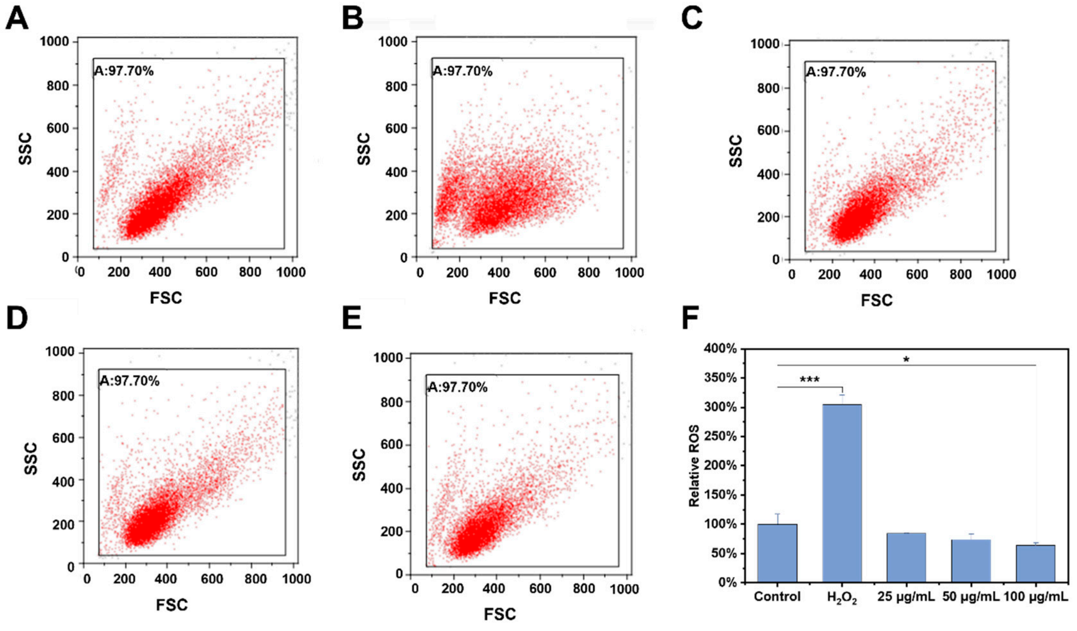

Figure S4. Representative flow cytometry histograms of DCF fluorescence intensity after 2 h of treatment with (A) Control, (B) 0.1mM H_2O_2, (C) 25 μg/mL CuO_2, (D) 50 μg/mL CuO_2, (E) 100 μg/mL CuO_2; (F) Quantitative analysis of mean fluorescence intensity. Data are presented as mean ± SD (n = 3) *(*P < 0.05, **P < 0.01, ***P < 0.001*).*
